# Prevalence of Malaria and Associated Knowledge, Attitude, and Practice among Suspected Patients in Bahir Dar Zuria District, Northwest Ethiopia

**DOI:** 10.1155/2021/3741413

**Published:** 2021-10-19

**Authors:** Zelalem Dejazmach, Getaneh Alemu, Mulat Yimer, Banchamlak Tegegne, Abel Getaneh

**Affiliations:** ^1^Department of Medical Laboratory Science, College of Health Sciences, Woldia University, Woldia, Ethiopia; ^2^Department of Medical Laboratory Science, College of Medicine and Health Sciences, Bahir Dar University, Bahir Dar, Ethiopia; ^3^Medical Parasitology, Amhara Public Health Institute, Bahir Dar, Ethiopia; ^4^Medical Parasitology and Vector Control, Bahir Dar University, Bahir Dar, Ethiopia

## Abstract

**Background:**

Control and prevention activities have brought substantial decline of malaria incidence in the last two decades in Ethiopia. However, lack of local data on the disease transmission and community knowledge, attitude, and practice about malaria are thought to reverse the trend of malaria in certain areas. Therefore, assessment of the prevalence and community awareness towards malaria plays pivotal role for the success of malaria control and prevention.

**Objective:**

To assess malaria prevalence and knowledge, attitude, and practice about malaria among febrile patients in Bahir Dar Zuria district, Northwest Ethiopia.

**Methods:**

A facility based crosssectional study was conducted from January to March 2020 among 149 febrile patients attending selected health centers in Bahir Dar Zuria district. Data about knowledge, attitude, and practice about malaria were collected using semistructured questionnaire. Blood sample from each participant was tested for *Plasmodium* species through malaria rapid diagnostic tests and blood film microscopy. Data were analyzed using statistical software for social sciences version 20.

**Results:**

Among 149 participants, 22 (14.8%) were positive for *Plasmodium* infection at least by one diagnostic methods. Prevalence of *P. falciparum* and *P. vivax* was 3.4% and 10.1%, respectively, while that of mixed infection was 1.3%. From the total study participants, 29.5% have good knowledge, 77.2% have positive attitude, and 34.9% have good practice towards malaria. Statistically significant associations were observed on knowledge with age group (*X*^2^ = 10.377, *P* = 0.035), educational level (*X*^2^ = 15.075, *P* = 0.001), family size (*X*^2^ = 7.601, *P* = 0.022), attitude level and practice level. Participants with family size < 5 were 6.841 (95% CI: 2.570-18.206, *P* ≤ 0.001) times more likely to have negative attitude as compared to those with family size ≥ 5.

**Conclusions:**

Prevalence of malaria in the study area was relatively high. Study participants had encouraging attitude; however, their knowledge and practice towards malaria were poor. Therefore, the existing malaria control activities should be supplemented with continuous health educations, aware the community, and ensure participation in the control and prevention activities.

## 1. Background

Malaria is a febrile disease caused by protozoan parasites of the genus *Plasmodium.* The disease remains one of the most severe public health problem worldwide, particularly in tropical and subtropical areas [1]. *Plasmodium (P) falciparum*, *P. vivax*, *P. malarae*, *P. ovale*, and *P. knowlesi* naturally infect human to cause malaria. However, *P. falciparum* and *P. vivax* are the most prevalent malaria parasites globally, the former being the most pathogenic [2].

In 2018, an estimated 228 million cases of malaria occurred leading to a death toll of 405, 000 worldwide [2]. Malaria remains to be the major public health problem in Ethiopia [3]. It is estimated that ~68% of the population is at risk of the disease and a point estimate of 2,362,979 cases and 4,757 deaths were reported in 2018 in the country [2]. *Plasmodium falciparum* and *P. vivax* are the two dominant species in Ethiopia which accounts 60% and 40% of malaria cases, respectively, and are prevalent in all malaria endemic areas. However, the proportion varies among geographical settings and season. In lowlands and during the major malaria transmission season, *P. falciparum* is much common while *P. vivax* is more common at higher altitudes and during the dry season [4]. Malaria is mainly seasonal, and transmission primarily occurs at altitudes below 2,000 meters above sea level in the country [5].

The disease is mainly transmitted through the bite of a female *Anopheles* mosquito [1], and the clinical symptoms are caused by asexual blood stages of the parasite [6]. The first signs and symptoms of malaria are nonspecific presenting with headache, fatigue, and abdominal discomfort followed by fever, perspiration, chills, and rigors. If left untreated, severe malaria develops which comprises of impaired consciousness, respiratory distress, metabolic acidosis, hypoglycemia, acute kidney injury, circulatory collapse, and pulmonary oedema [7]. Most severe and complicated manifestations and almost all malaria deaths are caused by *P. falciparum* [5].

Malaria-suspected patients should be confirmed by laboratory diagnosis and treated with efficacious antimalaria drugs within 24 hours of the onset of symptoms [8]. Artemisinin combination therapy and chloroquine are the first line drugs for the treatment of uncomplicated *P. falciparum* and *P. vivax* malaria, respectively, and intravenous artesunate or quinine therapy for complicated malaria [5]. Early diagnosis and immediate treatment is one of the main strategies in malaria prevention, control, and effective case management [8]. Moreover, there are also intervention strategies for malaria control activities that have brought substantial reduction of malaria incidence including environmental management, indoor residual spraying (IRS), and use of long-lasting insecticide-treated nets (ITNs) [1].

An updated data in the local prevalence of malaria helps not only to evaluate the local implementation of control and prevention activities but also to aware the healthcare providers and the community as well. On the other hand, many of the recently published epidemiological studies focus on asymptomatic participants by considering their role as potential sources of infection. However, data about the proportion of malaria patients among febrile cases helps to figure out the morbidity impact of the disease and for future planning.

Similarly, understanding knowledge level, perception, and practical behaviors of communities towards malaria is crucial in order to ensure appropriate intervention measures [9]. Different reports regarding the knowledge on malaria in different parts of Africa and around the world documented that there is a knowledge, attitude, and practice (KAP) gap among communities hindering people not to engage actively in the existing intervention programs [10]. People who have good KAP are believed to be in a better position to be protected against malaria [11, 12]. Findings of this study make inroads into the implementation of effective interventions in the area and indicate focus areas for enhancing community awareness and scaling up coverage of evidence-based interventions. Therefore, this study was conducted to assess prevalence of malaria and KAP among febrile patients in Bahir Dar Zuria district, Northwestern Ethiopia.

## 2. Methods

### 2.1. Study Design, Area, and Period

A facility-based crosssectional study was conducted in Yinesa, Andasa, and Robit health centers in Bahir Dar Zuria district, Northwest Ethiopia, from January to March 2020. The Bahir Dar Zuria district is one of the 14 districts of West Gojjam Zone, which is located at a distance of 560 km from capital city of the country, Addis Ababa, and the district is situated surrounding Bahir Dar city, capital of Amhara National Regional State. The altitude of the district ranges from 1700 to 2300 meters above sea level. The district receives an average annual rainfall of about 1035 mm. The minimum and maximum temperature lies at 10°C and 32°C, respectively [13]. The whole land mass of the district is classified as with the major and minor transmission seasons being September to December and April to May, respectively [14]. In the district, there are 9 health centers and 36 health posts. Both health centers and health posts provide diagnostic and treatment services to the community.

### 2.2. Sample Size Determination and Sampling Technique

The sample size was calculated using single population proportion formula based on the 95% confidence limits (Z*α*/2 = 1.96) and 5% margin of error (*d*) and previous prevalence (*p*) of 12.8% from Shiraro, North Ethiopia [15]. Sample size = (Z*α*/2)^2^ *p* (1 − *p*)/*d*^2^ = (1.96)^2^ (0.128) (1 − 0.128)/(0.05)^2^ = 172.

Among 9 health centers in the district, 3 were selected for data collection by simple random sampling technique. Accordingly, Andasa, Yinesa, and Robit health centers were selected. Number of study participants from each health center was allocated based on febrile patients flow during the same season of the previous year. Accordingly 50, 60, and 39 febrile patients were included from Andasa, Yinesa, and Robit health centers, respectively. Systematic random sampling was applied to select study participants from each health center. Clinically malaria suspected patients with age ≥ 15 who were sent to the laboratory for blood film examination and gave consent to participate in the study were included. Critically ill patients who were unable to respond to research questions and individuals who had taken antimalaria drugs within 4 weeks prior to data collection were excluded.

### 2.3. Data Collection

#### 2.3.1. Questionnaire Data

Amharic version of a semistructured questionnaire adapted from recent publications was used to collect data about the sociodemographic characteristics, knowledge, attitude, and practice of respondents related to malaria transmission, symptoms, and prevention methods. Questionnaire data were administered through face to face interview. Knowledge assessment questions were asked to each participant, and each correct response was given a score of 1 while a wrong or unsure response was scored 0. Total scores were interpreted based on Bloom's cut-off points (80.0–100.0% of correct responses meant a good knowledge, a score of 60.0–79.0% put a scorer in a level of satisfactory knowledge, and a poor knowledge was for the respondents with a score ≤ 59.0% of the correct responses were adapted and modified) [16]. Therefore, the scores with their respective knowledge levels were 5.6–7 good knowledge, 4.2–5.5 satisfactory knowledge, and <4.2 poor knowledge. Attitude was assessed by Likert's scale [17]. The questions on Likert's scale had positive and negative responses that ranged from agree (score 3), undecided (score 2), and disagree (score 1). The responses were summed up, and a total score was obtained for each respondent. The mean score was calculated and respondents with score of greater than or equal to the mean score (2.78) were considered as having positive attitude while those with score less than the mean score were taken as having negative attitude towards malaria. Practices were also determined using Likert's type. The scoring system of Likert's type scales with respect to respondents response ranging from never (score 0), sometimes (score 1), and always (score 2) were used and interpreted as good and poor practice based on the mean score [18].

#### 2.3.2. Blood Collection and Processing

Capillary blood sample was collected from each participant by finger puncture following standard procedure [5]. After removing the first drop, samples were used for thin and thick blood film preparation as well as for running malaria rapid diagnostic tests (RDTs). A multispecies care start RDT (Access Bio, Belgium) was used in the present study. Thick and thin blood smears for each participant were prepared on the same slide and labeled with sample number and date and let air dried. Then, thin blood smears were fixed with absolute methanol and allowed to air dry. Blood smears were stained with 10% Giemsa solution and examined with light microscope and reported following the standard protocols [5]. Identification code for each RDT device was given similar to the code used on the slide for each study participant. Then, 5 *μ*l of whole blood was added onto the test device window using the specimen transfer device (micropipette) provided with the kit, followed by adding 2 drops (60 *μ*l) of reagent buffer, and the result was recorded as per the instruction of the manufacturer.

### 2.4. Statistical Analysis

Data were checked for completeness and entered and analyzed in statistical package for social sciences (SPSS) version 20. Descriptive statistics was run to measure frequencies and percentages of the variables. Frequency distribution tables were used to quantify sociodemographic variables, knowledge, and attitude of respondents related to symptoms, causes, transmission, prevention, and control measures of malaria as well as practices toward malaria prevention and control methods. Associations between KAP and malaria infection were assessed through logistic regression. Factors associated with KAP level were also analyzed using odds ratio and chi-square. Variables with *P* value of <0.3 in the bivariable analysis were included in the multivariate logistic regression analysis. Variables with *P* value < 0.05 in the multivariable analysis were considered as statistically significant at 95% confidence level.

### 2.5. Ethics Approval and Consent to Participate

The present research was carried out after ethical approval was obtained from the Institutional Review Committee of College of Medicine and Health Sciences, Bahir Dar University with reference number CMHSc009/2020 on 03 January 2020. Additionally, supportive letters were obtained from Amhara Public Health Institute, West Gojjam Zone Health Department, and Bahir Dar Zuria district health office, and permission was obtained from each health center authorities. Informed verbal consent was obtained from each participant. Consent for participants with age 15-17 was taken in the presence of their parents/guardians. Verbal informed consent was acceptable and approved by the Bahir Dar University, College of Medicine and Health Sciences Research and Ethical Review Committee. Data on decisions of participants to agree or disagree was listed with an excel sheet by their code, and no personal identifier was included as participants were given a unique code. Malaria-suspected patients who were positive for malaria by any of the tests were linked to the respective health center for appropriate treatment.

## 3. Results

### 3.1. Sociodemographic Characteristics of Study Participants

We were unable to collect data from 172, as planned in the sample size calculation section, due to unprecedented COVID-19 outbreak at the time of data collection. Hence, data from 149 febrile patients was collected and included in the analysis. Among a total of 149 febrile patients participated in the present study, 89 (59.7%) and 60 (40.3%) were males and females, respectively. The participants' age ranged from 15 to 82 years old with mean age of 32.79 (±12.91 SD) years. All the study participants were rural inhabitants. Regarding to their educational status, 96 (64.4%) were unable to read while write, and the rest 53 (35.6%) were able to read and write or have attended formal education. More than half (53.7%) of malaria-suspected patients have history of fever within previous 1 year (Table 1).

### 3.2. Prevalence of *Plasmodium* Species

Among 149 participants, 20 (13.4%) and 19 (12.8%) were positive for *Plasmodium* infection as confirmed by RDTs and microscopy, respectively. Overall, 22 (14.8%) participants were positive by at least either of the diagnostic methods. Species level analysis revealed that the prevalence of *P. falciparum* and *P. vivax* was 3.4% and 10.1%, respectively, while that of mixed infection was 1.3%.

### 3.3. Knowledge of Respondents on Malaria

The study indicated that 144 (82.1%) respondents had heard about malaria. Nearly half of the respondents (48.3%) attributed the cause of malaria to mosquito bite and the remaining (49.7%) did not know the cause of malaria. More than half of respondents identified the major sign and symptoms of malaria correctly; 83 (55.7%), 83 (55.7%), and 94 (63.1%) mentioned fever, headache, and chills/shivering as symptoms of malaria, respectively. Fifty (33.6%) and 26 (17.4%) participants identified correctly under five children and pregnant women as the most susceptible segments of the population to malaria, respectively. In response to knowledge about prevention strategies, majority of the respondents (81.9%) mentioned that ITN as malaria preventive method while only 6 (4.0%) mentioned that malaria can be prevented using IRS. Only 44 (29.5%) participants responded that avoiding stagnant water prevents from malaria (Table 2).

Pearson's chi-square analyses revealed that age group (*x*^2^ = 10.377, *P* = 0.035) was significantly associated with participants' knowledge that proportion of good knowledge was highest among ≥36-year-old patients. Similarly, educational status, family size, attitude level, and practice level were also associated with participants' knowledge about malaria (Table 3).

### 3.4. Attitude towards Malaria and Health Seeking Behavior

From the total respondents, 130 (87.2%) agreed on seriousness and threat posed by malaria. The majority (88.6%) of respondents thought that malaria is curable disease. One hundred twenty-three (82.6%) participants agreed with the statement that malaria is preventable disease (Table 4). The majority (77.2%) of study participants had positive attitude while 34 (22.8%) had negative attitude towards malaria.

Statistically significant associations were observed between attitude level and family size that participants with family size < 5 were 6.841 (95% CI: 2.570-18.206, *P* ≤ 0.001) times more likely to have negative attitude as compared to those with family size ≥ 5. All other perceived factors were not associated with attitude level (Table 5).

### 3.5. Practices of Respondents towards Malaria Control and Prevention

Use of mosquito net was the most frequently applied method for malaria prevention by 117 (78.5%) respondents. Thirty-six (24.3%) respondents practiced compound/house sanitation as malaria preventive methods, and 25 (16.8%) did not practice for malaria prevention. The types of roof of almost all the respondents (98.0%) were corrugated iron and most (95.3%) of them owned ITNs (Table 6).

Statistically significant associations were observed between practice level and sex of participants. Males had around 52% less good practice as compared to females (Table 7).

## 4. Discussion

The overall malaria prevalence in the present study (14.8%) was in line with previous findings of 16.0% from Dilla Town, Southern Ethiopia [19], and results reported in north-western Tigray, Ethiopia (13.4%) [15]. However, malaria prevalence in the present study was higher than the study results from Shewa Robit, Ethiopia, (2.8%) [20]. The variation might be due to study design that the current study was facility based but the study conducted at Shewa Robit was community based. On the other hand, malaria prevalence in the present study was lower as compared to findings of 40.9% in Kola Diba [21], 25.0% in East Shewa [22] and 49.4% in Haro Limmu Woreda [23] all in Ethiopia. The low prevalence could be partly explained by the fact that the current data was collected during the dry (low malaria transmission) season while the previous studies were collected during the peak malaria transmission season in the country. Moreover, the difference might be due to variation in malaria endemicity. Another possible reason might be due to the effectiveness of malaria prevention and control strategies in Ethiopia. For example, the study conducted at Kola Diba was almost before 9 years but there have been many control and prevention strategies applied in the country such as increased bed net distribution and IRS.

In the current study, majority (96.6%) of the respondents had heard about malaria, which was comparable with previous study results of 97.6%-100.0% in Ethiopia [20, 24, 25]. However, it was higher than results in Kersa (85.9%) [26]. Seventy two (48.3%) participants implicated mosquito bite was the way of malaria transmission, similarly with results of 47.5% and 48.8% o from Assosa [9] and Tigray [27], respectively. On the other hand, it was lower than findings from Raya Azebo district (63%) [24], Shewa Robit (85.2%) [20], Tepi Town (86.7%) [25], Southern Ethiopia (83.7%) [28], Oromia Region (63.4%) [29], and Arba Minch (98.2%) [30]. It was also lower than findings from Nigeria (74.3%) [31], Tanzania (95.31%) [32], Iran (77.8%) [33], and Swaziland (92.8%) [34]. The difference might be due to variation of respondent characteristics like the educational level. In the present study, 64.4% of the respondents were illiterate, unlike those studies in Ethiopia (5.6%) [25] and Iran (37.3%) [33]. Sources of information might be the other reason for malaria transmission awareness difference, as about 92.6% of the respondents have no source of information in the last six months for malaria in our study. However, this study participants' knowledge about the means of transmission of malaria is much better than other studies conducted in Amhara region where 32.3% mentioned mosquito bite for transmission of malaria [35]. This variation might also be due to differences in educational level since 67.9% of the respondents in the previous study were illiterate.

More than half (55.0%) of the participants responded that main malaria transmission season was after main rainy season. Moreover, 49 (32.9%) responded during main rainy season and 12 (8.1%) responded all year round as the main malaria transmission season. Similar study conducted in Southern Ethiopia reported that respective proportion of participants responded that main malaria transmission season were cloudy weather (30.8%), rainy season (27.7%), dry season (32.7%), and any season (8.8%) [28].

Most of the respondents (91.3%) were familiar with at least one of the classical symptoms of malaria. More than half of respondents identified sign and symptoms of malaria correctly that 83 (55.7%), 83 (55.7%), and 94 (63.1%) mentioned fever, headache, and chills and shivering, respectively. Similar results were found from different KAP studies including Raya Azebo district [24] and Tigray region [27]. However, it was lower than findings from Shewa Robit where 94.4%, 84.5%, and 93.3% mentioned fever, headache, and chills and shivering, respectively [20]. Knowledge regarding malaria signs and symptoms was also lower than other reports in Ethiopia [9, 30] and Karachi [36]. On the contrary, it was higher than study results in Central Africa Republic that respondents had poor knowledge about malaria signs and symptoms [37].

In the present study, low level of awareness was observed with only 50 (33.6%) and 26 (17.4%) participants correctly identified under five children and pregnant women as the most susceptible groups for malaria infection, respectively. However, a high level of awareness was reported in other studies in Ethiopia where 85.1-90.3% and 59.0%-62.3% of respondents were aware that under 5 children and pregnant mothers are the most risk groups, respectively [25, 30].

With regard to knowledge in intervention measures for indoor prevention and vector control, 120 (81.9%) participants understood ITN as malaria preventive method. It was higher than reports from Myanmar that 65.6% of respondents mentioned sleeping with bed net as malaria preventive method [38]. However, it was lower when compared with two previous studies from Ethiopia with 95.4% [28] and 97.55% [39] of respondents agreed on the fact that use of ITN can prevent from malaria.

This study shows that 130 (87.6%) respondents agreed to seriousness and threat posed by malaria. It was in line with results from Raya Azebo district (90.8%) [24]. On the other hand, it was higher than the study findings in Tigray region (65.9%) [27]. One hundred twenty-three (82.6%) participants agreed with the statement that malaria is a preventable disease, which was comparable with the study conducted in Raya Azebo district where 87.9% respondents believed that malaria is a preventable disease [24]. However, it was lower than results from Shewa Robit where 90.58% respondents believed that malaria is a preventable disease [20]. On the contrary, it was higher than the study done in Tigray region (74.7%) [27] and Paksong district of Lao PDR (78%) [34].

A total of 44 (29.5%), 50 (33.6%), and 55 (36.9%) participants had good, satisfactory, and poor knowledge about malaria, respectively. This is lower when compared with a study from Southern Ethiopia, where 74.3% of respondents had good knowledge while the remaining 25.7% had poor knowledge [20]. It was also lower than the study conducted in Champasack Province of Lao PDR where 59.1% of respondents had good knowledge [40]. On the other hand, the knowledge level in the present study was higher as compared to previous findings from Mumbai. In Mumbai, 39.7%, 53.7%, and 5.8% had low, average, and high level of knowledge, respectively [41]. Differences in the overall civilization, health education and implementation coverage, health policy, and sociocultural habits are proposed factors for variations in knowledge level about malaria.

Statistically significant associations were observed on knowledge with age group, educational level, family size, attitude level, and practice level. Participants of older age group, i.e., ≥36, possessed significantly high percentage of good knowledge (47.7%) as compared to other age groups, 15–25 (27.3%) and 26–35 (25.0%). Likewise, respondents who can read and write and above had significantly high frequency of good knowledge (59.1%) than those who were unable to read and write (40.9%). The participants whose family size was ≥5 had higher proportion of good knowledge (65.9%) as compared to family members were <5 (34.1%). Moreover, the respondents who had positive attitude have high percentage of good knowledge (93.2%) than who had negative attitude towards malaria (6.8%). Finally, those who had poor practice had higher frequency of good knowledge (63.6%) than those who had poor practice level (36.4%).

This study revealed that 115 (77.2%) participants had positive attitude while 34 (22.8%) had negative attitude towards malaria in terms of its seriousness or threat, prevention and control. This is lower when compared with studies in Karachi where 97% of the respondents had good attitude [36]. To the contrary, it was higher than the study from Amhara National Regional State of Ethiopia with 69% respondents having positive attitude towards malaria [35]. Moreover, it was higher than the study conducted in Mumbai, that 16.9% of respondents had positive attitude [41]. In the present study, participants who had <5 family size were 6.8 times more positive attitude compared to those with ≥5 family size.

Result observed in this study revealed that 52 (34.9%) participants had good and 97 (65.1%) had poor practice towards malaria prevention and control measures. This is lower when compared to studies in Southern Ethiopia [9], Hadya zone [12], and Karachi [36] where 67.7%, 32.3%, and 59% of the study participants had good practice, respectively. Conversely, studies from LAO PDR reported that only 5.7% had good practice [40]. Variations in knowledge about malaria, availability of intervention tools, attention given by health authorities, and local transmission level of the disease might bring differences in the attitude and practice levels about malaria. Male had around 52% less good practice as compared to female. The limitation of this study is that we used nonvalidated questionnaire adapted from recent similar studies [9–12, 20, 25, 27] and translated to Amharic language. We recommend implementation of community health education focusing on malaria transmission, health and economic outcome, intervention approaches, and how people can involve in intervention activities in the local context.

## 5. Conclusions

The current study illustrated that prevalence of malaria among febrile patients in the study area was high with relative dominance of *P. vivax*. It has also been indicated that study participants had encouraging attitudes. Nonetheless, inadequate knowledge about malaria transmission and prevention as well as poor practice towards malaria control and prevention was noticed in the present study.

## Figures and Tables

**Table 1:** Sociodemographic characteristics and clinical data of febrile patients in Bahir Dar Zuria district, Northwest Ethiopia, from January to March 2020.

Characteristics	Categories	Frequency	Percentage
Sex	Male	89	59.7
	Female	60	40.3

Age group (in years)	15-25	55	36.9
	26-35	36	24.2
	36-45	38	25.5
	≥46	20	13.4

Educational level	Unable to read and write	96	64.4
	Able to read and write	12	8.1
	Primary	24	16.1
	Secondary and above	17	11.4

Primary occupation	Farmer	124	83.2
	Nonfarmer	25	16.8

Marital status	Single	30	20.1
	Married	119	79.1

Family size	<5	75	50.3
	≥5	74	49.7

History of fever within previous 1 year	Yes	69	46.3
	No	80	53.7

Causes of previous fever	Malaria	26	37.7
	Other	17	24.6
	Do not know	26	37.7

**Table 2:** Knowledge of respondents regarding to cause, sign and symptoms, transmission, and preventive methods of malaria, Bahir Dar Zuria district, Northwest Ethiopia, 2020.

Variables	Category	Frequency (*N* = 149)	Percentage
Do you know malaria	Yes	144	96.6
	No	5	3.4

Causes of malaria	Mosquito bite	72	48.3
	Cold temperature	2	1.3
	Poor sanitation	1	0.7
	Unknown	74	49.7

Main malaria transmission seasons^∗^	Before main rainy season	9	6.0
	Main rainy season	49	32.9
	After main rainy season	82	55.0
	All year round	12	8.1
	Unknown	6	4.0

The main sign and symptoms of malaria^∗^	Fever/sweat	83	55.7
	Headache	83	55.7
	Feeling cold	94	63.1
	Chills/shivers	94	63.1
	Nausea/vomiting	20	13.4
	Weakness, joint/muscle pain	38	25.5
	Loss of appetite	46	30.9

How do people get malaria?^∗^	Mosquitoes bite	72	48.3
	Drinking dirty water	52	34.9
	Working in sun	10	6.7
	Did not get enough food	16	10.7
	Leave near collected water	33	22.1
	Working in cold temperature	20	13.4
	I do not know	35	23.5

Which group of people is more affected by malaria?^∗^	All adult (age > 15)	3	2.0
	Children (5-15 years old)	2	1.3
	Children <5 years old	50	33.6
	Pregnant women	26	17.4
	All are equally affected	78	52.3
	I do not know	8	5.4

What are the prevention methods of malaria? ^∗^	Using bed net	120	81.9
	Draining stagnant water	44	29.5
	Spraying house with chemicals	6	4.0
	Sanitation	2	1.3
	Unknown	25	16.8

^∗^More than one answer is possible.

**Table 3:** Crosstabulation of chi-square analysis of association of knowledge with sociodemography, among malaria-suspected cases in Bahir Dar Zuria district, Northwest Ethiopia, 2020.

Characteristics	Categories	Knowledge level			*X* ^2^	*P* value
		Poor (%)	Satisfied (%)	Good (%)		
Sex	Male	32 (58.2)	29 (58)	28 (63.6)	0.820	0.396
	Female	23 (41.8)	21 (42)	16 (36.4)		

Age group (in years)	15-25	29 (52.7)	14 (28)	12 (27.3)	10.377	0.035
	26-35	9 (16.4)	16 (32)	11 (25)		
	≥36	17 (30.9)	20 (40)	21 (47.7)		

Educational level	Unable to read and write	41 (74.5)	37 (74)	18 (40.9)	15.075	0.001
	Able to read and write and above	14 (25.5)	13 (26)	26 (59.1)		

Primary occupation	Farmer	47 (85.5)	45 (90)	32 (72.7)	5.312	0.07
	Nonfarmer	8 (14.5)	5 (10)	12 (27.3)		

Marital status	Single	13 (23.6)	7 (14)	10 (22.7)	1.774	0.412
	Married	42 (76.4)	43 (86)	34 (77.3)		

Family size	<5	34 (61.8)	26 (52)	15 (34.1)	7.601	0.022
	≥5	21 (38.2)	24 (48)	29 (65.9)		

**Table 4:** Attitude towards malaria and health seeking behavior and information access of the respondents in Bahir Dar Zuria district, Northwest Ethiopia, 2020.

Variables	Category	Frequency (*N* = 149)	Percentage
Do you think that malaria is public health problem?	Yes	130	87.2
	No	10	6.7
	I do not know	9	6.0

Do you think that malaria is curable?	Yes	132	88.6
	No	12	8.1
	I do not know	5	3.4

Is malaria preventable?	Yes	123	82.6
	No	16	10.7
	I do not know	10	6.7

Duration of the present fever/illness (in hours)	≤24	41	38.9
	>24	108	61.1

Reasons for delay after fever started	Too far	8	7.4
	Cannot afford cost of transport	6	5.6
	I go if got sever	84	77.8
	Health facility was closed	10	9.3

What did you do first when this fever started	Went to health facility	11	7.4
	Use herb/natural remedies	9	6
	Went to religious leaders	8	5.4
	Went drug store to get drug	5	3.4
	We have done nothing	116	77.9

Sought treatment before visiting the health center	Yes	12	8.1
	No	137	91.9

Health education on malaria in the last 6 months	Yes	11	7.4
	No	138	92.6

**Table 5:** Multivariate logistic regression analysis of attitude level among febrile patients in Bahir Dar Zuria district, Northwest Ethiopia, 2020.

Characteristics	Categories	Attitude level		COR (95% CI)	*P* value	AOR 95% CI	*P* value
		Negative (%)	Positive (%)				
Sex	Male	22 (64.7)	67 (58.3)	1.313 (0.593-2.909	0.502	0.458 (0.184-1.136)	0.092
	Female	12 (35.3)	48 (41.3)	1			

Age group (in years)	15-25	17 (50)	38 (33.0)	0.583 (0.248-1.371)	0.216	0.851 (0.315-2.300)	0.750
	26-35	5 (14.7)	31 (27.0)	1.617 (0.518-5.049)	0.408	2.244 (0.660-7.629)	0.196
	≥36	12 (35.3)	46 (40.0)	1			

Educational level	Unable to read and write	23 (67.6)	73 (63.5)	1.203 (0.534-2.711)	0.656		
	Able to read and write and above	11 (32.4)	42 (36.5)	1			

Primary occupation	Farmer	26 (76.5)	98 (85.2)	1.774 (0.689-4.564)	0.235	1.514 (0.503-4.562)	0.461
	Nonfarmer	8 (23.5)	17 (14.8)	1			

Marital status	Single	7 (20.6)	23 (20.0)	0.964 (0.373-2.490)	0.940		
	Married	27 (79.4)	92 (80.0)	1			

Family size	<5	27 (79.4)	48 (41.7)	5.384 (2.167-13.378)	≤0.001	6.841 (2.570-18.206)	≤0.001
	≥5	7 (20.6)	67 (58.3)	1			

**Table 6:** Practices of respondents towards malaria prevention and control strategies in Bahir Dar Zuria district, northwest Ethiopia, 2020.

Variables	Category	Frequency (*N* = 149)	Percentage
What do you do in your household to prevent malaria?^∗^	Using mosquito nets	117	78.5
	Spray house with DDT	5	3.4
	Compound/house sanitation	36	24.2
	Close door and window early night	6	4.0
	Nothing	25	16.8

Type of roof	Thatched	3	2.0
	Corrugated iron	146	98.0

Household ownership of ITNs	Yes	142	95.3
	No	7	4.7

Number of ITNs owned	**1**	**11**	7.7
	**2**	**35**	24.6
	**3**	**58**	40.8
	**≥4**	**38**	26.8

Frequency of night slept under ITNs in the last 15 days	All nights	52	36.6
	Sometimes	13	9.2
	Only few nights	15	10.6
	None of the nights	62	43.7

**Table 7:** Multivariate logistic regression analysis of practice level among malaria-suspected cases in Bahir Dar Zuria district, Northwest Ethiopia, 2020.

Characteristics	Categories	Practice level		COR (95% CI)	*P* value	COR (95% CI)	*P* value
		Poor (%)	Good (%)				
Sex	Male	64 (71.9)	25 (28.1)	0.477 (0.240-0.949)	0.035	0.476 (0.231-0.980)	0.044
	Female	33 (55.0)	27 (45.0)	1			

Age group (in years)	15-25	41 (74.5)	14 (25.5)	0.484 (0.217-1.077)	0.075	0.531 (0.201-1.398)	0.200
	26-35	22 (61.1)	14 (38.9)	0.902 (0.385-2.109)	0.811	0.838 (0.348-2.015)	0.693
	≥36	34 (58.6)	24 (41.4)	1			

Educational level	Unable to read and write	61 (63.5)	35 (36.5)	1.215 (0.597-2.474)	0.591		
	Able to read and write and above	36 (67.9)	17 (32.1)	1			

Primary occupation	Farmer	80 (64.5)	44 (35.5)	1.169 (0.467-2.925)	0.739		
	Nonfarmer	17 (68.0)	8 (32.0)	1			

Marital status	Single	24()	6 (11.5)	0.397 (0.151-1.044)	0.061	0.667 (0.210-2.182)	0.514
	Married	73 (75.3)	46 (88.5)	1			

Family size	<5	48 (49.5)	27 (51.9)	1.102 (0.562-2.163)	0.777		
	≥5	49 (50.5)	25 (48.1)	1			

## Data Availability

The authors confirm that all data underlying the findings are fully available without restriction. All relevant data are within the manuscript.

## Abbreviations

IRS: Indoor residual spraying
ITNs: Long-lasting insecticide-treated nets
KAP: Knowledge, attitude, and practice
RDT: Rapid diagnostic tests.
